# Comparative Analysis of Circulating and Synovial Immune Cells in Early Untreated Rheumatoid Arthritis and Their Relationship With Molecular Pathology and Disease Outcomes

**DOI:** 10.1002/art.43194

**Published:** 2025-07-07

**Authors:** Felice Rivellese, Elena Pontarini, Liliane Fossati‐Jimack, Rita A. Moura, Vasco C. Romão, João Eurico Fonseca, Alessandra Nerviani, Cankut Çubuk, Katriona Goldmann, Giovanni Giorli, Myles Lewis, Michele Bombardieri, Costantino Pitzalis, the Pathobiology of Early Arthritis Cohort (PEAC) investigators

**Affiliations:** ^1^ Centre for Experimental Medicine and Rheumatology, William Harvey Research Institute, Queen Mary University of London and Barts Health NHS Trust and Barts Biomedical Research Centre, NIHR London United Kingdom; ^2^ Centre for Experimental Medicine and Rheumatology, William Harvey Research Institute, Queen Mary University of London London United Kingdom; ^3^ Instituto de Medicina Molecular João Lobo Antunes, Faculdade de Medicina, Universidade de Lisboa Lisbon Academic Medical Center Lisbon Portugal; ^4^ Instituto de Medicina Molecular João Lobo Antunes, Faculdade de Medicina, Universidade de Lisboa and Department of Rheumatology, Hospital de Santa Maria, Centro Hospitalar Universitário Lisboa Norte, Lisbon Academic Medical Center Lisbon Portugal; ^5^ Centre for Experimental Medicine and Rheumatology, William Harvey Research Institute, Queen Mary University of London and Barts Health NHS Trust and Barts Biomedical Research Centre, NIHR, London, United Kingdom, and Department of Biomedical Sciences Humanitas University and IRCCS Humanitas Research Hospital Milan Italy

## Abstract

**Objective:**

To assess the relationship of circulating and synovial immune cells with synovial molecular pathology and disease outcomes in patients with early rheumatoid arthritis (RA).

**Methods:**

Patients with early (<12 months) treatment‐naive RA (n = 144) from the Pathobiology of Early Arthritis Cohort were included in this post hoc analysis. Following ultrasound (US)‐guided synovial biopsy of the most active joint, patients received standard of care treatment and were observed for 12 months. Synovial biopsies were analyzed by immunohistochemistry and classified into synovial pathotypes. Peripheral blood (PB) samples (n = 70) underwent flow cytometry, whereas RNA sequencing from synovial tissue (n = 144) and matched PB (n = 55) were analyzed using signature‐based deconvolution.

**Results:**

T peripheral helper cells (Tph) cells, identified by flow cytometry, were the only circulating immune cell subset positively correlated with both systemic markers of inflammation (erythrocyte sedimentation rate and C‐reactive protein) and local inflammation (US synovial thickening and synovitis score). Circulating Tph cells were also significantly higher in patients who were seropositive and patients with lymphomyeloid synovitis. Conversely, circulating B cells showed a significant inverse correlation with markers of inflammation, US scores, and disease activity. Signature‐based deconvolution of matched synovial and PB identified divergent immune cell signatures. Although PB signatures showed no associations with longitudinal outcomes, synovium signatures were linked to clinical outcomes. In particular, patients achieving remission at six months (Disease Activity Score in 28 joints < 2.6) had higher baseline synovial Tph signatures and a greater posttreatment reduction of the Tph signature.

**Conclusion:**

Patients with early untreated RA showed divergent immune cell signatures between synovia and PB. Tph cells, in both compartments, emerged as key markers for inflammation, disease activity, and treatment response.

## INTRODUCTION

Rheumatoid arthritis (RA) is characterized by clinical heterogeneity, mirrored by the diverse infiltration of immune cells within the synovial tissue.^1^ Alterations of immune cells T and B cells,^2^ monocytes,^3^ dendritic cells,^4^, ^5^ and natural killer (NK) cells^6^, ^7^, ^8^ have been observed in the peripheral blood (PB) of patients with RA. Although circulating immune cells have been associated with disease activity and disease outcomes, including treatment response, their usage as biomarkers remains unvalidated. For example, an expansion of PB memory B cells has been noted in patients with RA, even in early disease stages.^9^, ^10^ Increased pretreatment PB memory B cells have been identified in patients responding to B cell‐depleting therapies.^11^ However, consistent B cell depletion after rituximab, regardless of treatment response, limits the predictive value of B cell assessment before and after treatment.^12^ Similarly, other circulating immune cell patterns, including monocytes, dendritic cells, and T cells, fail to predict treatment response. Thus, despite the established role of immune cells in RA pathogenesis, the relationship between their circulating levels and disease outcomes remains uncertain. In some cases, changes in circulating immune cell frequency may reflect a general inflammatory response rather than disease‐specific immunophenotypes. Conversely, the direct study of the synovial membrane has revealed distinct cellular and molecular signatures linked to disease activity and response to conventional synthetic disease modifying antirheumatic drugs (csDMARDs).^13^, ^14^ Similarly, in patients with established RA, synovial B cell lineage signatures have been identified as a promising marker of disease progression and treatment response,^15^, ^16^ and, more recently, in‐depth histologic and molecular analyses of synovial biopsies identified immune response gene signatures associated with response to rituximab and tocilizumab, and a stromal and fibroblast signature in patients refractory to all medications.^17^ The analysis of the synovial tissue by mass cytometry and single‐cell transcriptomics led to the identification of a T helper cell subpopulation, named T peripheral helper (Tph) cells.^18^ These cells were found to be expanded in the PB of patients with active seropositive RA and reduced by treatment.^19^ However, because of the small sample size, their precise association with treatment response remains unclear. Furthermore, mass cytometry analyses have revealed 18 unique cell populations in the synovia of patients with RA, including subsets of monocytes, fibroblasts, and B and T cells.^20^ However, the association of these subsets with clinical outcomes remains to be elucidated.

Therefore, in this study, we aim to establish the relationship between circulating immune cells and infiltrating synovial immune cells through in‐depth cellular and molecular analyses of matched PB and synovial tissue in a unique cohort of patients with early treatment‐naive RA. The study incorporated matched blood and synovial tissue biopsies obtained before any potential treatment modification of the disease. Our findings underscore Tph cells as clinically relevant cellular signatures linked to lymphomyeloid synovitis and are indicative of disease progression and treatment response.

## PATIENTS AND METHODS

### Patients

144 patients fulfilling the 2010 American College of Rheumatology/EULAR classification criteria for RA^21^ consecutively recruited in the Pathobiology of Early Arthritis Cohort (PEAC) at Barts Health NHS Trust^22^ between 2009 and 2019 were selected for this post hoc analysis (see Supplementary Table 1 for patient demographics and baseline characteristics). Inclusion criteria for the PEAC study were: (1) the presence of at least one swollen joint amenable to synovial biopsy (ultrasound [US] synovial thickening >1); (2) symptoms duration less than 12 months; (3) naive to csDMARD or steroid treatments. Following a baseline synovial biopsy (see below for details), patients were observed for 12 months and underwent quarterly assessments performed by clinical research fellows (rheumatologist trainees) or rheumatology consultants, with the treatment given according to the standard of care following a treat‐to‐target approach, that is, all patients started methotrexate unless contraindicated, were escalated to a second csDMARD if the target was not met at follow‐up and to a biologic DMARD as per UK National Institute for Health and Clinical Excellence guidelines in place at the time of the study (after treatment with two csDMARDs and Disease Activity Score in 28 joints [DAS28] >5.1). Routine blood was processed by an NHS laboratory, whereas study blood taken in parallel was processed for peripheral blood mononuclear cell (PBMC) isolation and flow cytometry by the Centre for Experimental Medicine and Rheumatology laboratory. Plain radiographs of the hands and feet performed at baseline and 12‐month follow‐up were scored in a time sequential order according to the van der Heijde‐modified total Sharp score (mTSS) by a single reader blinded to all clinical or histologic data. Progression at 12 months was defined as any increase in the mTSS (delta mTSS ≥1). All patients gave written informed consent, and the study received local ethics approval (REC ref 05/Q0703/198).

### Synovial biopsy

All patients underwent US‐guided synovial biopsies of a clinically active joint selected according to a previously reported algorithm defining the level of synovial tissue thickness aimed at confirming localized synovitis while ensuring good synovial tissue retrieval.^23^ From each procedure, a minimum of six synovial samples were retained for subsequent histologic analysis and six for RNA extraction. In parallel, blood samples were collected, and PBMCs were isolated using gradient separation with Lymphoprep (StemCell Tecnologies).

### Histopathological scoring

Formalin‐fixed, paraffin embedded sections (3μm) underwent standard hematoxylin and eosin and immunohistochemistry staining (CD20, CD68, CD3, CD138). Following semiquantitative assessment (0–4), according to previously validated score,^24^ biopsies were then stratified into one of the three synovial pathotypes, as previously described^13^: (1) lymphomyeloid presence of grades 2–3—CD20+ aggregates, (CD20 ≥2) and/or CD138 ≥2; (2) diffuse‐myeloid—CD68 sublining (SL) ≥2, CD20 ≤1 and/or CD3 ≥1, CD138 ≤2; and (3) pauci‐immune‐fibroid—CD68 SL <2 and CD3, CD20, CD138 <1.

### Flow cytometry

Flow cytometry was performed on cryopreserved PBMCs (n = 70 patients). Cells were thawed by adding dropwise warm medium (RPMI‐10% FCS), washed once and resuspended in phosphate buffered saline. Upon thawing, cells were counted, and viability was verified using trypan blue staining (median viability 86%, with 82% of the samples showing viability >70%).

Dead cells were excluded by live‐dead exclusion dye (Aqua Zombie, BioLegend); FcReceptors were blocked using an Fc receptor‐blocking solution (TruStain, BioLegend). Cells were stained with four different panels (Supplementary Table 2, also indicating the number of samples analyzed for each panel), according to standard procedures. Samples were acquired on an LSRII Fortessa cytometer (BD Biosciences) equipped with 405, 488, 561, and 640 nm lasers, and analyzed using FlowJo version 10 (FlowJo LLC).

### RNA extraction and RNA sequencing

RNA extraction and RNA sequencing were performed as previously described.^22^ Briefly, RNA was extracted from synovial tissue homogenized at 4°C in Trizol (ThermoFisher Scientific), observed a phenol/chloroform extraction. 1μg of total RNA was used as input material for library preparation using TruSeq RNA Sample Preparation Kit version 2 (Illumina). Generated libraries were amplified with 10 cycles of polymerase chain reaction. The libraries were first multiplexed (five per lane) and then sequenced on Illumina HiSeq2500 (Illumina) to generate 50 million paired ends with 75 base pair reads. As for blood, whole blood samples were preserved in RNALater solution (ThermoFisher Scientific) at 500 mL whole blood to 1.3 mL RNALater solution and stored at 80°C before extraction. Blood samples in RNALater solution were thawed on ice and RNA was prepared using the Ambion Ribo‐Pure Blood kit (ThermoFisher Scientific), as per the manufacturer's instructions. The concentration and purity of RNA samples was measured using the NanoDrop 2000C (Lab Tech) and RNA quality (RIN) was assessed by Agilent 2100 Bioanalyser (Agilent Technologies) and 2200 TapeStation (Agilent Technologies).

### RNA sequencing analysis and deconvolution

RNA sequencing was analyzed as previously described.^14^ Following the identification of outliers using principal component analysis (3 patients for synovium, 0 for blood as described in Cell Report), the remaining samples were deconvoluted using ImmuneDeconv.^25^ Publicly available single‐cell expression data with cell subset annotations were downloaded from ImmPort (https://www.immport.org/shared/study/SDY998). For each cell subset, we collapsed normalized single‐cell expression profiles into artificial‐bulk count matrices by summing the counts for each gene using bulk_construct function of the MuSiC R package, and then the bulk dataset was subjected to quantile normalization using preprocessCore (https://github.com/bmbolstad/preprocessCore). The normalized artificial‐bulk data was deconvoluted with five different deconvolution tools (xCell, MCP‐counter, quanTIseq, EPIC, and Cibersort) for their performance comparison. The cell fractions estimated by these tools were visualized by heatmap plots. The concordance between cell subset annotations and in silico cell type predictions was explored manually to assess their performance.

For the enrichment of single cell states, we scored each subtype using a modular approach that integrates gene signatures^26^ obtained from differential gene expression analysis and known markers that are previously described by scRNA‐Seq study.^20^ Module scores for each subtype were calculated using *AddGeneSetScore*,^27^ a repurposing of the AddModuleScore function from the R package Seurat,^28^ adapted for bulk RNA sequencing data processed with DESeq2 (https://doi.org/10.1186/s13059-014-0550-8). After comparing module scores using top five, top 10, and top 20 differentially expressed genes, we used the top five exclusively differentially expressed genes (based on area under the curve scores) as subtype‐specific gene sets because this allowed us to differentiate a higher number of subsets. Module scores between responders and nonresponders were compared using the Wilcoxon test. ComplexHeatmap was used for clustering.^29^

### Statistical analysis

Measures of central tendency, dispersion, and statistical tests are indicated in each figure legend. Unless stated otherwise, the following statistical tests have been used: Mann‐Whitney U test for comparison between two groups; Kruskal‐Wallis test with Dunn's post hoc test for multiple group comparison; chi‐square test for proportions; Spearman's test for correlations. Statistical analyses were performed using R (version 4.2.1 or later). *P* < 0.05 was considered statistically significant. Correction for multiplicity was applied using the false discovery rate method in correlation matrices involving multiple comparisons between cell subsets and various clinical/biologic parameters. Figure legends specify when multiple testing correction was applied.

## RESULTS

### Relationship of circulating immune cells with systemic inflammation and disease activity

**Figure 1:**
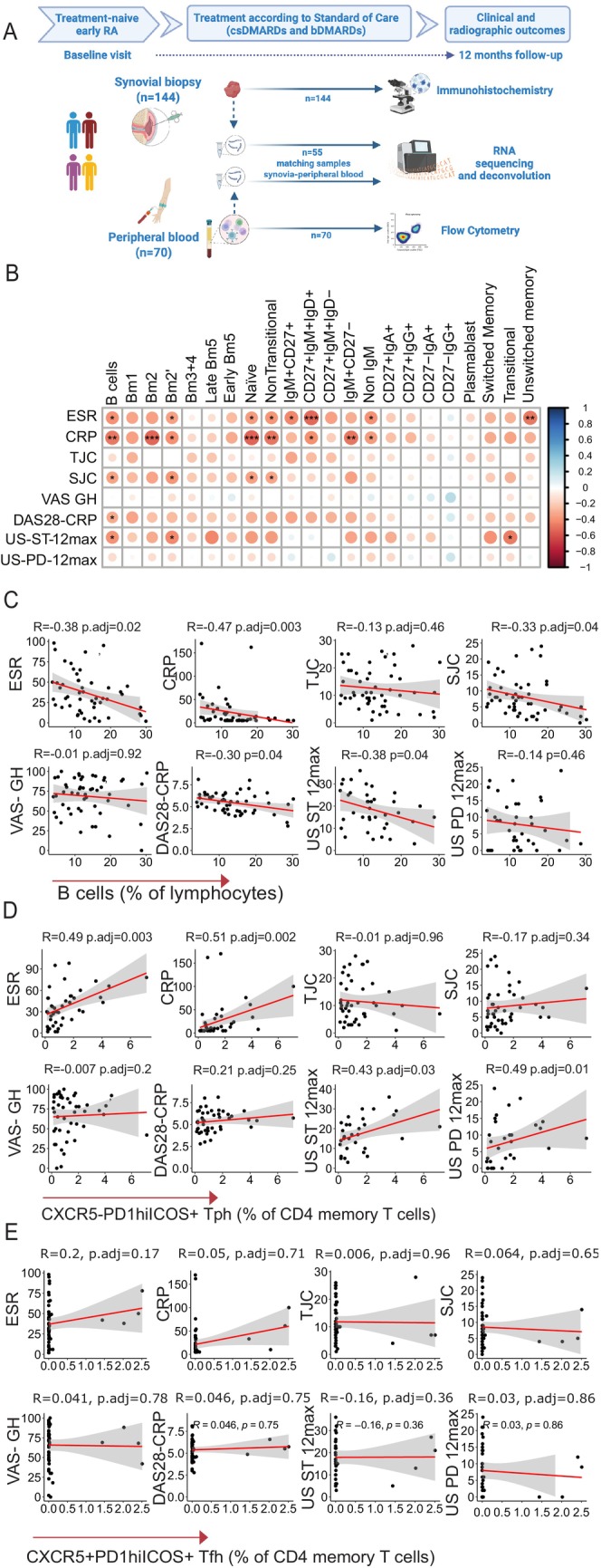
Correlation of circulating immune cell subsets with disease activity. Pictured are correlations between immune cell subsets assessed by flow cytometry and markers of inflammation, DAS28s and their components, and US scores. (A) Overview of samples included in the analysis, including synovial biopsies (n = 144) and matched peripheral blood (n = 70), undergoing immunohistochemistry and flow cytometry. RNA extracted from synovial tissue (n = 144) and peripheral blood mononuclear cells (n = 55) underwent RNA sequencing. (B) Correlation matrix for B cell subsets identified by flow cytometry and markers of disease activity; dot size and color intensity corresponding to the correlation coefficient R, with positive correlations in blue and negative correlations in red, using Spearman correlation, with correction for multiple comparisons by false discovery rate. (C–E) Individual correlation plots for B cells, Tph cells, and Tfh cells, with Spearman correlation coefficients (R) and adjusted *P* values (Spearman correlation with false discovery rate correction). *P < 0.05; **P < 0.01; ***P < 0.001. adj, adjusted; bDMARD, biologic disease‐modifying antirheumatic drug; Bm, mature B cells; CRP, C‐reactive protein; csDMARD, conventional synthetic disease‐modifying antirheumatic drug; CXCR, CXC chemokine receptors; DAS28, Disease Activity Score in 28 joints; ESR, erythrocyte sedimentation rate; RA, rheumatoid arthritis; SJC, swollen joint count; TJC, tender joint count; Tfh, T follicular helper; Tph, T peripheral helper; US, ultrasound; US‐ST 12, ultrasound synovial thickening score in 12 joints; US‐PD 12, ultrasound power doppler score in 12 joints; VAS‐GH, visual analog scale global health (patient‐reported).

To explore the association of circulating immune cells with clinical phenotypes and histologic and molecular profiles, we used flow cytometry to analyze the frequency of immune cell subsets to include B cells, T cells, monocytes, NK cells, and regulatory T cells in the PB of patients with early treatment‐naive RA recruited to the PEAC study, thus with available matched synovial tissue, as shown in Figure 1A. Details of the gating strategy are provided in Supplementary Figure 1.

First, we examined the correlation of circulating immune cells with systemic inflammation and disease activity. B cells and their subsets showed significant inverse correlations, persisting after correction for multiple testing, with markers of inflammation (erythrocyte sedimentation rate [ESR], C‐reactive protein [CRP]), swollen joint count, DAS28 scores, and US scores (Figure 1B and C). As for T cells, CD8^+^GNZB^+^CD69B^+^ T cells showed positive correlations with markers of inflammation and disease activity scores (Supplementary Figure 2A). Conversely, monocytes, NK cells, and Treg cells did not exhibit any significant correlations (Supplementary Figure 2B and C). Next, we looked at CXC chemokine receptor (CXCR) 5^−^PD1^hi^ inducible costimulator positive CD4^+^ T peripheral helper cells (Tph) cells, a subset known to be expanded in the PB of patients with RA.^19^ Accordingly, Tph showed a significant positive correlation with markers of inflammation (ESR and CRP, R = 0.49, *P* < 0.001 and R = 0.51, adjusted *P* < 0.001, respectively) and US scores (synovial thickening, R = 0.43, adjusted *P* = 0.015) (Supplementary Figure 2D). Importantly, this correlation was not evident for the CXCR5^+^ counterpart, T follicular helper cells (Tfh) (Supplementary Figure 2D).

**Figure 2:**
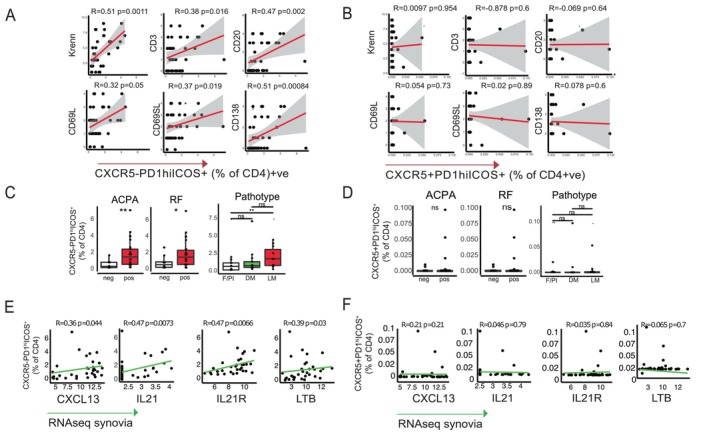
Circulating Tph and Tfh cells. Correlation plots of circulating (A) Tph and (B) Tfh cells with synovial semiquantitative immune scores Krenn total synovitis score (0–9 semiquantitative scores measured with hematoxylin and eosin), CD3 T cells, CD20 B cells, CD68 lining and sublining macrophages, and CD138 plasma cells (0–4 semiquantitative scores by immunohistochemistry). R = Spearman's rank correlation coefficient, with exact (nominal) *P* values. n = 51 samples measured in Figure 2A and B. (C) Tph and (D) Tfh cells, measured by flow cytometry, in patients stratified according to the positivity of ACPA, RF, and according to synovial pathotypes. Mann‐Whitney test was used. n = 48 for ACPA and RF (antibody status missing for three patients); n = 50 for histology (1 sample ungraded). Correlation plots of circulating (E) Tph and (F) Tfh cells with representative lymphoid genes measured by RNA sequencing (seq). R = Spearman's rank correlation coefficient, with exact (nominal) *P* values. n = 32 samples measured in Figure 2E and F. *P < 0.05; **P < 0.01. ACPA, anti–citrullinated protein antibody; CXCL, CXC chemokine ligands; CXCR, CXC chemokine receptors; IL, interleukin; LTB, lymphotoxin β; RF, rheumatoid factor; Tfh, T follicular helper; Tph, T peripheral helper. Color figure can be viewed in the online issue, which is available at http://onlinelibrary.wiley.com/doi/10.1002/art.43194/abstract.

Next, we investigated the relationship of circulating immune cells with synovial immune cell infiltration in matched PB and synovial biopsies (Figure 1A). Although the majority of the circulating subsets did not show any correlation with synovial histology scores (Supplementary Figure 2E–H), a notable exception was observed for Tph cells, displaying significant positive correlations with immune cell infiltration in synovia (Figure 2A). This association was not observed for Tfh (Figure 2B). Additionally, circulating Tph cells were significantly higher in patients with positive anti–citrullinated protein antibody or rheumatoid factor, and, in line with their correlation with immune cell infiltration in synovia, were significantly higher in patients with a lymphomyeloid pathotype, characterized by the infiltration of T and B cells (Figure 2C and D). Accordingly, circulating Tph cells were significantly correlated with key genes for Tph cell biology and known to be involved in the formation of ectopic lymphoid structures (ELS), such as CXC chemokine ligand (CXCL) 13, interleukin (IL) 21 and IL21R, and lymphotoxin β (Figure 2E and F).

### Benchmarking transcriptome deconvolution in PB and synovial tissue against flow cytometry and histopathology

Next, we aimed to use bulk transcriptome deconvolution to dissect the characteristics of immune cells infiltrating the synovial tissue. To this aim, we benchmarked the performance of five commonly used tools, primarily developed in cancer research, in the blood and synovial tissue of patients with RA. In the PB, the tools showed various degrees of correlation with their counterparts measured by flow cytometry, which served as our gold standard. Interestingly, all tools performed quite poorly for certain cell types, such as CD4 T cells, with MCPcounter and xCell showing the most favorable correlations across multiple subsets (Supplementary Figure 3A).

Next, we applied deconvolution to the synovial transcriptome, using semiquantitative scores by immunohistochemistry as a surrogate gold standard (Supplementary Figure 3B). As seen for PB, MCPcounter and xCell showed the highest correlation rates.

Taking into account these results, our analyses continued with xCell because this tool can identify up to 39 immune cells, providing a significant advantage over traditional histology, although with the caveat of poor performance for certain cell populations, as shown for CD4 T cells.

### Matched PB and synovial transcriptome deconvolution identify divergent associations with histopathological and clinical features

**Figure 3:**
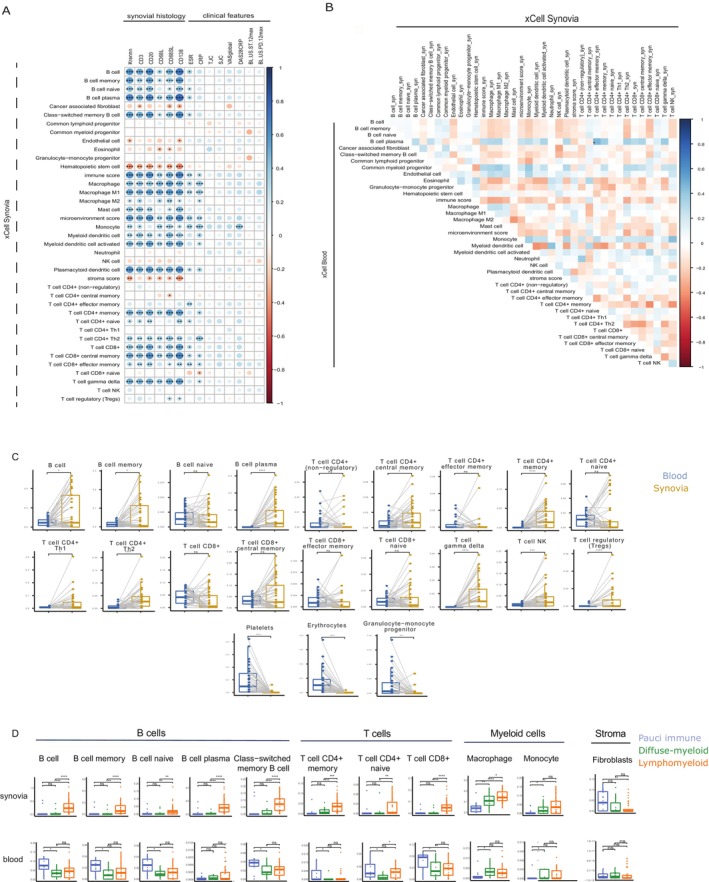
Deconvolution of synovial and peripheral blood RNA sequencing. Deconvolution applied to RNA sequencing of RNA extracted from synovial tissue biopsies (n = 49) and peripheral blood (n = 36). (A) Matrix showing the correlation between xCell signatures in synovial tissue and synovial histology/clinical features. Dot size corresponds to Spearman correlation coefficients (−1/+1), shown in a color scale, with positive correlations in blue and negative correlations in red. Spearman correlation was used with correction for multiple comparison by false discovery rate. (B) Matrix showing the correlation between peripheral blood and synovial tissue signatures; color‐coded Spearman correlation coefficients were used, as in A. (C) xCell immune cell signatures in matched peripheral blood (blue) and synovial samples (yellow). Mann‐Whitney test was used, n = 36. (D) Immune cell signatures in patients stratified according to synovial pathotypes in synovia (top) and peripheral blood (bottom). Kruskal‐Wallis with Dunn's post hoc test was used with 49 synovial tissue samples and 36 peripheral blood samples. Individual dots represent individual patients, boxplots show median and first and third quartiles, and whiskers extend to the highest and lowest values. *P < 0.05; **P < 0.01; ***P < 0.001. CRP, C‐reactive protein; DAS28, Disease Activity Score in 28 joints; ESR, erythrocyte sedimentation rate; NK, natural killer; SJC, swollen joint count; Th, T helper; TJC, tender joint count; US‐PD 12, ultrasound power doppler score in 12 joints; US‐ST 12, ultrasound synovial thickening score in 12 joints; VAS‐GH, visual analog scale global health (patient‐reported). Color figure can be viewed in the online issue, which is available at http://onlinelibrary.wiley.com/doi/10.1002/art.43194/abstract.

To further explore the presence and relevance of immune cells in matched PB and synovial tissue, we used xCell to deconvolute blood and synovia bulk transcriptome available from the same patients (see Figure 1A for an overview of the samples used). In synovia, signatures for B cells, plasma cells, T cells, and macrophages showed significant positive correlations with histologic semiquantitative scores (Figure 3A), whereas fibroblasts, hematopoietic stem cells, and stromal scores had negative correlations with synovial immune cell infiltration assessed by histology (Figure 3A). Synovial macrophage/monocyte signatures were the only cell signatures to show a significant correlation with disease activity scores (Figure 3A). When comparing immune cell signatures in matched PB and synovial samples, although no significant correlations were found, most cells showed a clear trend toward negative correlations, which suggests the same cell types might have opposing trends in the two compartments (Figure 3B).

Upon stratifying patients by synovial pathotypes, synovial B and T cell signatures were higher in patients with a lymphomyeloid pathotype, as expected for a pathotype characterized by the infiltration of B and T cells. On the contrary, circulating B cells and total CD4+ and CD8+ T cell signatures were lower in patients with synovial lymphomyeloid pathotypes. On the other hand, lymphomyeloid and diffuse‐myeloid pathotypes, both characterized by the infiltration of macrophages, showed higher myeloid signatures in both PB and synovial tissue.

Overall, we observed a potential divergence of immune cell signatures between PB and diseased tissue, emphasizing how the PB may not always accurately reflect synovial inflammation, thus reinforcing the relevance of studying the diseased tissue.

### Module signatures of synovial single cell states define diverse disease phenotypes

**Figure 4:**
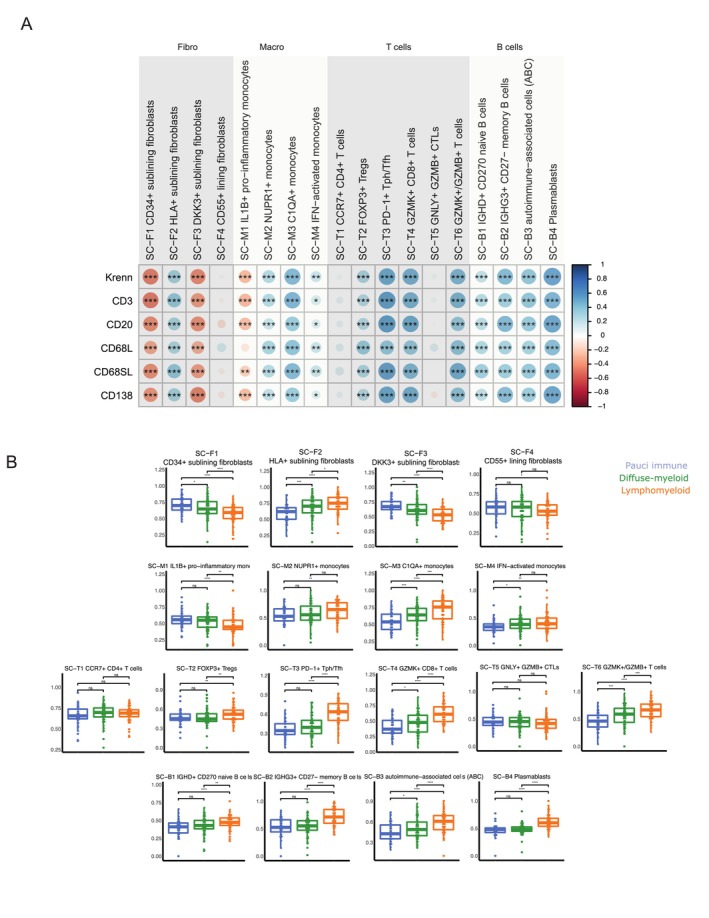
Single cell state signatures and synovial inflammation. (A) Matrix showing the correlation of single cell (SC) state signatures with synovial inflammation. Dot size corresponds to Spearman correlation coefficients as shown in color scale, with positive correlations in blue and negative correlations in red (−1/+1), n = 49. Spearman correlation with correction for multiple comparison by false discovery rate was used. (B) Single cell state signatures in patients stratified according to synovial pathotypes. Kruskal‐Wallis with Dunn's post hoc test was used with 49 synovial tissue samples and 36 peripheral blood samples. Individual dots represent individual patients, boxplots show median and first and third quartiles, and whiskers extend to the highest and lowest values. *P < 0.05, **P < 0.01, ***P < 0.001 CRP, C‐reactive protein; DAS28, Disease Activity Score in 28 joints; DKK, Dickkopf‐related protein; ESR, erythrocyte sedimentation rate; GZMB, granzyme B; GZMK, granzyme K; HLA, human leukocyte antigen; IFN, interferon; IL, interleukin; SJC, swollen joint count; Tfh, T follicular helper; TJC, tender joint count; Tph, T peripheral helper. Color figure can be viewed in the online issue, which is available at http://onlinelibrary.wiley.com/doi/10.1002/art.43194/abstract.

**Figure 5:**
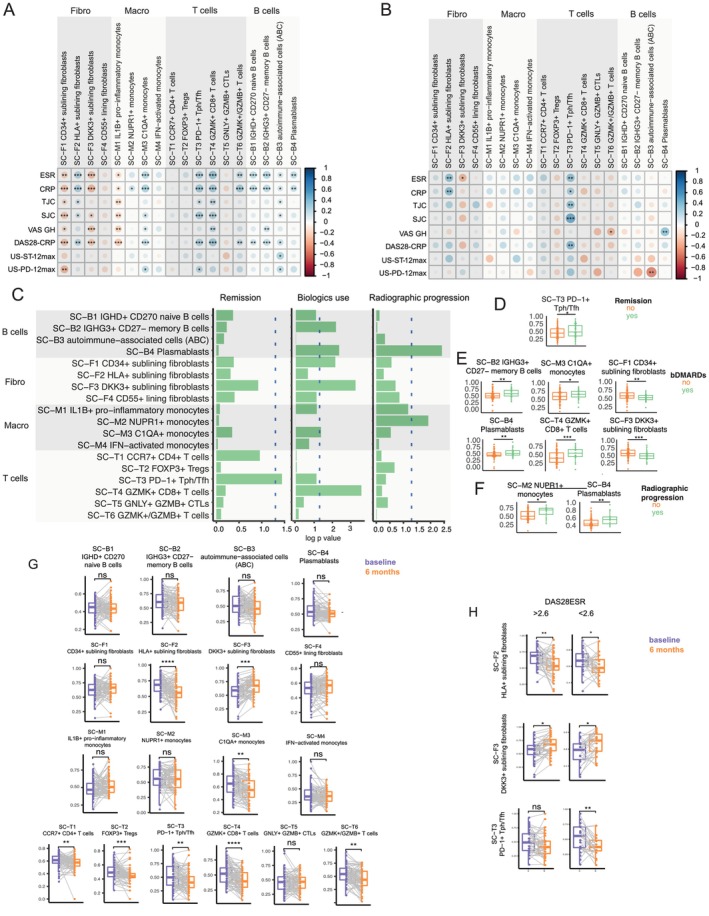
Single cell state signatures and disease outcomes. Matrix showing the correlation of single cell state signatures in (A) synovial tissue and (B) peripheral blood with DAS and their components and ultrasound scores. Dot size corresponds to Spearman correlation coefficients (−1/+1), as shown in color scale, with positive correlations in blue and negative correlations in red. Spearman correlation was used with correction for multiple comparison by false discovery rate. (C) Comparison of baseline synovial immune cell signatures in patients stratified according to remission at six months (DAS28 CRP <2.6), biologic treatment at 12 months, and radiographic progression at 12 months (delta van der Heijde‐modified total Sharp score (mTSS) ≥ 1). Lines show *P* values, with dotted line corresponding to *P* < 0.05, Mann‐Whitney. n = 121 for DAS28 at six months, n =107 for treatment with biologic drugs at 12 months, and n = 96 for radiographic progression at 12 months. (D–F) Individual dot plots showing baseline synovial immune cell signatures significantly different as in C. Mann‐Whitney test was used. The values for n are as in Figure 5C. Individual dots represent individual patients, boxplots show median and first and third quartiles, and whiskers extend to the highest and lowest values. (G) Single cell scores in matched baseline (before treatment) and synovial biopsies at six months (after treatment with csDMARDs). (H) Significantly different single cell scores in patients stratified according to DAS28 at six months less than or greater than 2.6. The Mann‐Whitney test was used. *P < 0.05, **P < 0.01, ***P < 0.001. bDMARD, biologic disease‐modifying antirheumatic drug; CCR, C‐C chemokine receptor type; CRP, C‐reactive protein; csDMARD, conventional synthetic disease modifying antirheumatic drug; DAS28, Disease Activity Score in 28 joints; DKK, Dickkopf‐related protein; ESR, erythrocyte sedimentation rate; GZMB, granzyme B; GZMK, granzyme K; HLA, human leukocyte antigen; IGHD, immunoglobulin heavy constant delta; IGHG3, immunoglobulin heavy constant gamma 3; IL, interleukin; NUPR, nuclear protein 1; SJC, swollen joint count; Tfh, T follicular helper; Tph, T peripheral helper; TJC, tender joint count; US‐ST 12, ultrasound synovial thickening score in 12 joints; US‐PD 12, ultrasound power doppler score in 12 joints; VAS‐GH, visual analog scale global health (patient‐reported). Color figure can be viewed in the online issue, which is available at http://onlinelibrary.wiley.com/doi/10.1002/art.43194/abstract.

Next, as we aimed to investigate synovium‐specific immune cell subsets described by mass cytometry and single cell transcriptomic,^20^ we used their top five expressed genes as a proxy to assess their presence in the matched bulk synovial and peripheral transcriptome h (Supplementary Figure 4A). Tissue‐resident cells, such as fibroblast and macrophages subsets were significantly higher in synovia, while most T and B cell subsets were higher in PB (Supplementary Figure 5B). Notably, however, PD1+ Tph/Tfh cells, granzyme K (GZMK) positive/GNZMB+ T cells, and plasmablast signatures were higher in synovia, aligning with their known role in driving synovial inflammation in patients with RA (Supplementary Figure 5B). We then examined the correlations of these signatures with synovial inflammation. Unsurprisingly, given the divergence we observed between synovial and blood signatures, none of the synovial single cell signatures showed any correlation with either synovial inflammation (data not shown) On the contrary, in synovia, CD34+ sublining fibroblasts, Dickkopf‐related protein 3 (DKK3)‐positive sublining fibroblasts, and IL‐1B+ pro‐inflammatory macrophages inversely correlated with immune cell scores, whereas other subsets showed positive correlations (Figure 4A). When stratifying patients by synovial pathotypes, specific signatures were associated with each pathotype (Figure 4B). Specifically, CD34+ sublining fibroblasts, DKK3+ sublining fibroblasts and CD55+ lining fibroblasts were significantly higher in patients with a pauci‐immune pathotype, supporting the idea that stromal cells might drive inflammation in this subset of patients. Macrophage signatures were higher in both myeloid and lymphomyeloid pathotypes, except for IL‐1B+ macrophages, which were up‐regulated in patients classified with fibroid or pauci‐immune pathotypes. Finally, most T and B cell subsets were up‐regulated in patients with lymphomyeloid pathotypes, consistent with the pathotype's characteristic abundance of B and T cells. Next, we assessed the associations of single cell signatures with clinical disease activity (Figure 5A and B). Synovial signatures for CD34+ sublining fibroblasts, DKK3+ sublining fibroblasts, and IL‐1B+ pro‐inflammatory macrophages negatively correlated with markers of inflammation and disease activity, whereas C1QA+ macrophages, PD1+ Tph T cells GZMK+CD8+ T cells and all B cells had positive correlations. In contrast, in circulation, a positive correlation was only observed for PD1+ Tph/Tfh cells (Figure 5B), in line with the flow cytometry results in which circulating CXCR5− Tph cells emerged as markers of inflammation and disease activity.

To explore the impact of these signatures on disease outcomes, we assessed baseline synovial single cell modules in association with different outcomes: (1) remission as determined by DAS28 (score < 2.6) at six months; (2) progression to biologic DMARDs within one year; and (3) any worsening of radiographic scores at one year (mTSS ≥1) (Figure 5C).

The Tph/Tfh signature was uniquely linked to treatment response (Figure 5D). Patients progressing to biologic DMARD treatments exhibited elevated baseline memory B cells, plasmablasts, C1QA+ monocytes, and GZMK+ CD8 T cells, whereas CD34+ and DKK3+ fibroblast signatures were lower (Figure 5E). In addition, patients with signs of radiographic progression (delta mTSS ≥1) had increased baseline plasmablast and nuclear protein 1 positive monocyte (Figure 5F). When examining the changes in repeat synovial biopsies after six months of csDMARD treatment (Figure 5G), significant reductions were observed in C1QA+ monocytes and various T cell subsets, including PD1+ Tph cells. Significant reductions were also observed in pro‐inflammatory human leukocyte antigen‐positive fibroblasts, whereas DKK3+ fibroblasts increased posttreatment. Changes in fibroblasts were consistent regardless of six month remission status, whereas Tph reduction occurred only in patients achieving DAS28 remission at 6 months (Figure 5H). Importantly, single cell signatures in blood did not show any significant association with disease outcomes (data not shown), including Tph/Tfh signatures. Overall, these findings suggest that pretreatment synovial single cell modular signatures are linked to disease outcomes, including treatment response, treatment with biologic DMARDs, and structural damage progression.

## DISCUSSION

Although numerous associations between circulating immune cells and clinical outcomes have been reported in patients with RA, it has been difficult to consistently relate them to clinical outcomes and treatment response. On the other hand, recent studies analyzing the molecular pathology of synovial biopsies have reported specific signatures linked to prognosis (structural damage) and treatment response.^15^, ^16^, ^17^, ^30^ Therefore, in this study, we aimed to explore the relationship of PB immune cells with the cellular and molecular profile of the synovial tissue in a biopsy‐driven cohort with early untreated. We observed inverse correlations between circulating B cells and markers of inflammation, disease activity, and US scores, aligning with previous findings of lower B cell counts in patients with active RA,^31^ including patients with very early RA^9^, ^32^ and patients with arthralgia.^33^ Treatment with biologics has been shown to restore the frequency of circulating memory B cells,^10^, ^11^, ^34^ and low levels of pretreatment B cells have been associated with lower chances of response to antitumor necrosis factor.^35^ Here, we expanded these observations defining that the low B cell count extends to specific subsets, including memory B cells.^36^, ^37^ Although the role of B cells in RA pathogenesis is well established and the effectiveness of B cell‐depleting therapies in patients with RA is well‐known, the usage of circulating B cells as predictive markers remains limited. Even though pretreatment levels of specific CD27+ memory B cells^11^, ^38^ and preplasma cells^39^ have been associated with response to rituximab, their use in clinical practice as predictive markers is inadequate. At least in part, this is probably linked to the uncertainty of whether circulating immune cells reflect the inflammatory burden in the synovial tissue. Our results define the relationship between PB and synovia, highlighting the importance of studying both circulating and synovial immune cells to establish the link between circulatory and synovial cells. Upon studying the synovial tissue, pretreatment levels of B cells and posttreatment changes have been linked to treatment response to B cell depletion ^40^, emphasizing the potential clinical relevance of understanding these dynamics. Recently, two large multicenter randomized biopsy‐driven clinical trials have provided promising insights into the ability of synovial signatures to predict disease outcomes,^15^, ^16^ whereas deep molecular phenotyping has identified specific signatures associated with multidrug resistance.^17^

Here, we used matched synovial tissue and PB samples to study the relationship of circulating immune cells, assessed by flow cytometry, and synovial inflammation. Most immune cells, including total CD4 and CD8 T cells, B cells, Treg cells, monocytes, and NK cells, showed no correlations. Notably, on the other hand, Tph cells, previously identified in synovial tissue and circulation,^19^, ^41^ emerged as a subset associated with both systemic and synovial inflammation. Circulating Tph cells were elevated in patients with seropositive RA and active disease, particularly in those with a lymphomyeloid synovial pathotype characterized by infiltrating B and T cells. This establishes circulating Tph cells as peripheral biomarkers for lymphomyeloid synovitis, a finding particularly relevant given the scarcity of peripheral biomarkers associated with this subtype of synovial inflammation because the only other circulating biomarker ever found in association with lymphomyeloid synovial inflammation and ELS formation is CXCL13,^42^ a chemokine produced mainly by Tph cells and their CXCR5+ counterpart.

Regarding molecular investigations, previous transcriptomic analyses of the PB in patients with early RA identified very few differentially expressed genes compared with the synovial tissue.^14^ Leveraging the availability of matched PB and synovial whole transcriptomes, we employed deconvolution tools to analyze both tissues from the same patients. As most of the existing deconvolution tools were developed in cancer tissues and have not been tested or validated in other diseases, we benchmarked the performance of five such tools using flow cytometry in the PB and immunohistochemistry in the synovial tissue as gold standards. xCell was selected for its ability to estimate the infiltration of up to 39 immune cell subsets, expanding our assessment compared to conventional histology. Notably, Cibersort,^43^ a widely used tool in oncology, demonstrated relatively poorer performance in both PB and synovial tissue of patients with RA compared with other tools.

By using deconvolution with xCell on matched synovial and PB samples, we uncovered diverging relationships between circulating immune cell subsets and synovial inflammation/pathotypes. Specifically, patients classified as lymphomyeloid, with abundant lymphoid cell infiltration in synovia, exhibited significantly lower levels of B and T cell signatures in circulation. Conversely, those with myeloid infiltration in the synovia consistently showed higher levels of myeloid cells in both synovial tissue and PB. These results, linked with the flow cytometry data indicating inverse correlations of B cells with disease activity, suggest a possible explanation for why PB analysis often falls short in identifying clinically useful biomarkers, emphasizing the importance of directly studying the diseased tissue.

In recent years, advancements in mass cytometry and single cell transcriptomic analyses have enhanced our understanding of synovial tissue in patients with RA, revealing 18 unique cell subsets/states across fibroblast, monocyte/macrophage, B, and T cell lineages.^20^ However, the relevance of these subsets in PB and their associations with clinical outcomes remained unclear. Analyzing single cell signature modules in synovial and PB transcriptomes based on synovial pathotypes, we found up‐regulated fibroblast subsets, including the DKK3+ subset, in the synovium of patients with a pauci‐immune/fibroid pathotype. The DKK3+ fibroblast signature was also elevated in patients with multidrug resistant or refractory RA.^17^ Fibroblast subsets showed negative correlations with disease activity, as did IL‐1B+ pro‐inflammatory macrophages, previously linked to leukocyte‐rich RA. Our study compared three synovial RA pathotypes, revealing lower IL1B+ macrophage signatures in lymphomyeloid compared to diffuse‐myeloid and pauci‐immune pathotypes, providing further insights into the synovial immune landscape.

When examining single cell module signatures in the PB, we observed significant correlations with synovial inflammation and disease activity scores only for PD1+ Tph cells. This aligns with our flow cytometry findings, reinforcing the notion that PD1+ Tph cells serve as PB markers of synovial inflammation and disease activity.

Finally, our study revealed an association of pretreatment synovial signatures with disease outcomes, including treatment response, use of biologic DMARDs and progression of structural damage. Specifically, PD1+ Tph cells were associated with DMARD response, suggesting their potential as PB predictors for both disease activity and treatment response because this is in line with previous results showing a reduction of Tph levels following treatment with csDMARDs.^19^ Additionally, the use of biologic DMARDs correlated with elevated levels of immune cell states, including memory B cells, plasmablasts, C1QA+ monocytes, and GZMK+ CD8 T cells. Conversely, fibroblast states were lower in patients progressing to biologics, consistent with our prior findings associating DKK3+ fibroblasts with refractory RA.^17^ Lastly, plasmablasts were linked to radiographic progression, supporting prior observations identifying a plasmablast signature as a predictor of structural damage.^14^, ^22^

This study has some limitations. First, this is a post hoc analysis without a predetermined statistical plan, therefore the study has an exploratory nature. Second, although we analyze a unique cohort of patients with early, treatment‐naive RA with matched synovial and PB samples, the sample size is relatively limited, so our findings warrant further investigations in larger cohorts. The relatively limited sample size meant we could not correct for potential confounders such as age, body mass index, and gender. Third, it is crucial to note that deconvolution tools estimate relative cell abundance, not absolute values, and they struggle with heavily overlapping cell signatures, thus performing quite poorly in estimating specific cell subsets, as observed for CD4 T cells. Therefore, when attempting to estimate the presence of synovium‐specific cell states, we opted to use the top differentially expressed genes as a proxy to estimate their presence. Even though these signatures do not provide exact subset proportions, they revealed significant associations with disease outcomes that are pathogenetically and clinically relevant, warranting exploration in future studies. Finally, more advanced single cells analyses have recently led to the identification of 77 synovial cell states, with patients then clustered into seven synovial Cell Type Abundance Phenotypes (CTAPs) were associated with disease outcomes, including treatment response/nonresponse.^42^ The relevance of these subsets in circulation vs synovia and the possibility of reproducing the CTAP classification using blood signatures remains to be investigated. Even though our results highlight the difficulties in identifying circulating signatures that can accurately mirror synovial inflammation, the pursuit of a liquid biopsy for inflammatory arthritis continues to be an ongoing challenge, which can be hopefully addressed by the combined study of PB and synovia.

In conclusion, our results highlight the importance of synovial tissue analyses in identifying clinically relevant associations between immune cells and disease outcomes, with particular reference to pretreatment cell state signatures among which Tph cells emerge as potential circulating biomarkers of lymphomyeloid synovitis and disease activity and showing promise as predictive markers for treatment response.

## AUTHOR CONTRIBUTIONS

All authors contributed to at least one of the following manuscript preparation roles: conceptualization AND/OR methodology, software, investigation, formal analysis, data curation, visualization, and validation AND drafting or reviewing/editing the final draft. As corresponding author, Prof Pitzalis confirms that all authors have provided the final approval of the version to be published and takes responsibility for the affirmations regarding article submission (eg, not under consideration by another journal), the integrity of the data presented, and the statements regarding compliance with institutional review board/Declaration of Helsinki requirements.

## Supporting information


**Disclosure form**.

**Appendix S1:** Supplementary Information
