# Supplementary material for: Comparative Analysis of Circulating and Synovial Immune Cells in Early Untreated Rheumatoid Arthritis and Their Relationship With Molecular Pathology and Disease Outcomes
Source: Arthritis Rheumatol. 2025 Jul 7;77(10):1349–61. doi: 10.1002/art.43194 (PMC12479182; doi:10.1002/art.43194)
Supplement: Supplementary file 2 — Appendix S1: Supplementary Information [file ART-77-1349-s001.docx]

**Supplementary material to Rivellese, Pontarini et al .**

Relationship Between Circulating and Synovial Immune Cells and Their Impact on Disease Outcomes in Early Untreated Rheumatoid Arthritis

**Supplementary Table 1.** Patient characteristics

|  | **Early RA**  n=144 |
| --- | --- |
| **Age** years, mean (SD) | 51.1 (15.4) |
| **Female/male** % | 70.1% |
| **DAS28** mean (SD) | 5.37 (1.13) |
| **TJ** mean (SD) | 11.7 (7.5) |
| **SJ** mean (SD) | 7.8 (5.2) |
| **VAS GH** mean (SD) | 64.5 (24.8) |
| **ESR** mm/h mean (SD) | 39.8 (28.2) |
| **CRP** mg/L mean (SD) | 22.3 (28.3) |
| **ACPA-positive, %** | 75 |
| **RF-positive, %** | 72.9 |

DAS28 Disease Activity Score 28 joints; TJ Tender Joints; SJ Swollen Joints; VAS GH Visuo-Analogic Score Global Health; ACPA Anti Citrullinated Protein Antibodies measured by clinically available standard path-lab CCP2 assay; RF Rheumatoid Factor

**Supplementary Table 2. Antibodies used for flow cytometry panels**

**Supplementary Figure_1. Gating Strategies**

**Supplementary Figure_2 Correlation histology**

Matrix showing the correlation between immune cell subsets assessed by flow cytometry and disease activity scores (A-D) and synovial semi-quantitative immune scores (E-H): Krenn total synovitis score (0-9 semiquantitative scores by hematoxylin and eosin); CD3 T cells, CD20 B cells, CD68 lining and sublining macrophages and CD138 plasmacells (0-4 semiquantitative scores by immunohistochemistry). A and F, T cell subsets; B and G, monocytes; E, B cell subsets; C and H, T regulatory cells; D, T peripheral helper cells and T follicular helper cells.

Dot size corresponding to R and *p<0.05, **p<0.01, ***p<0.001, Spearman correlation, with correction for multiple comparison by False Discovery Rate (FDR)

n=70 in A and F; n= 52 in E; n=62 in B and G; n= 51 in C,D and H.

**Supplementary figure_3 "Benchmarking against flow cytometry and synovial histology**

**
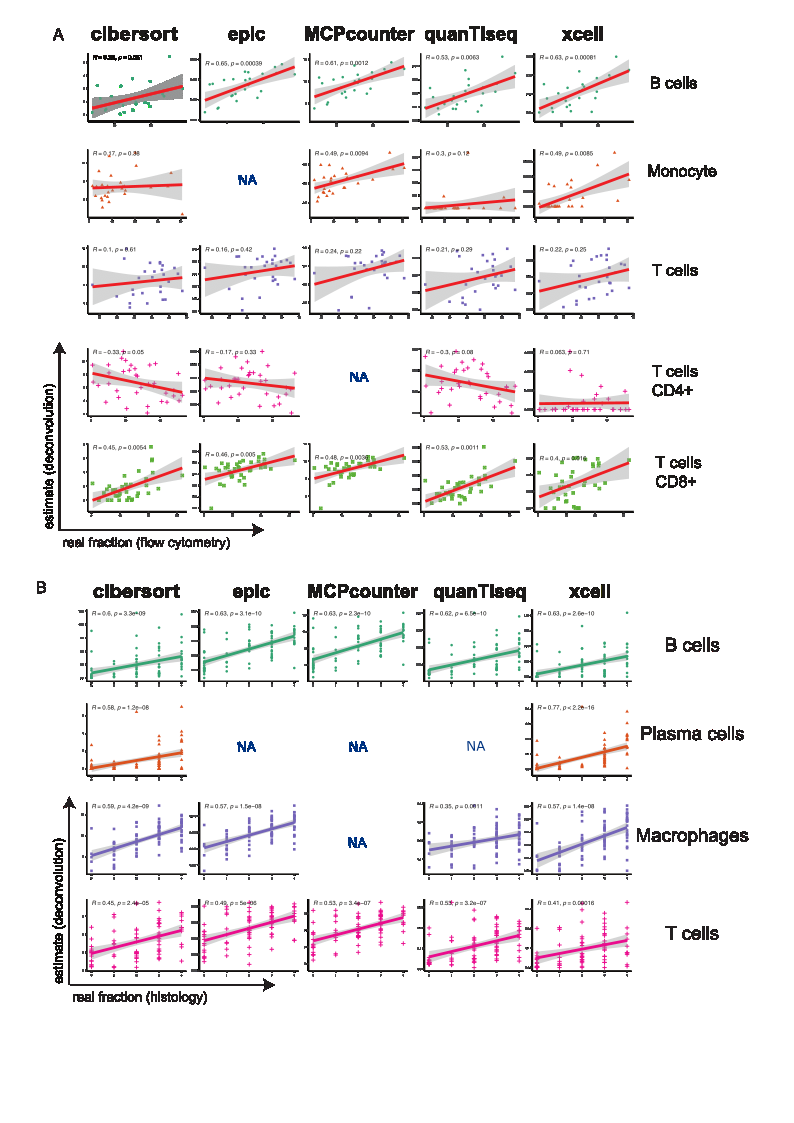
**

a, plots showing the correlation between RNAsequencing deconvolution signatures in the peripheral blood, using the deconvolution tool specified on top, and the same cell assessed by flow cytometry (as gold standard).

b, plots showing the correlation between RNAsequencing deconvolution signatures in the synovial tissue, using the deconvolution tool specified on top, and synovial semi-quantitative scoes by histology (as surrogate gold standard).

R= Spearman's rank correlation coefficient, with exact (nominal) p values. n=51 in a and n=32 in b. Empty plots correspond to cell types not available for the specific tool.

**Supplementary Figure_4 Single cell state signatures in peripheral blood and synovia.**

**
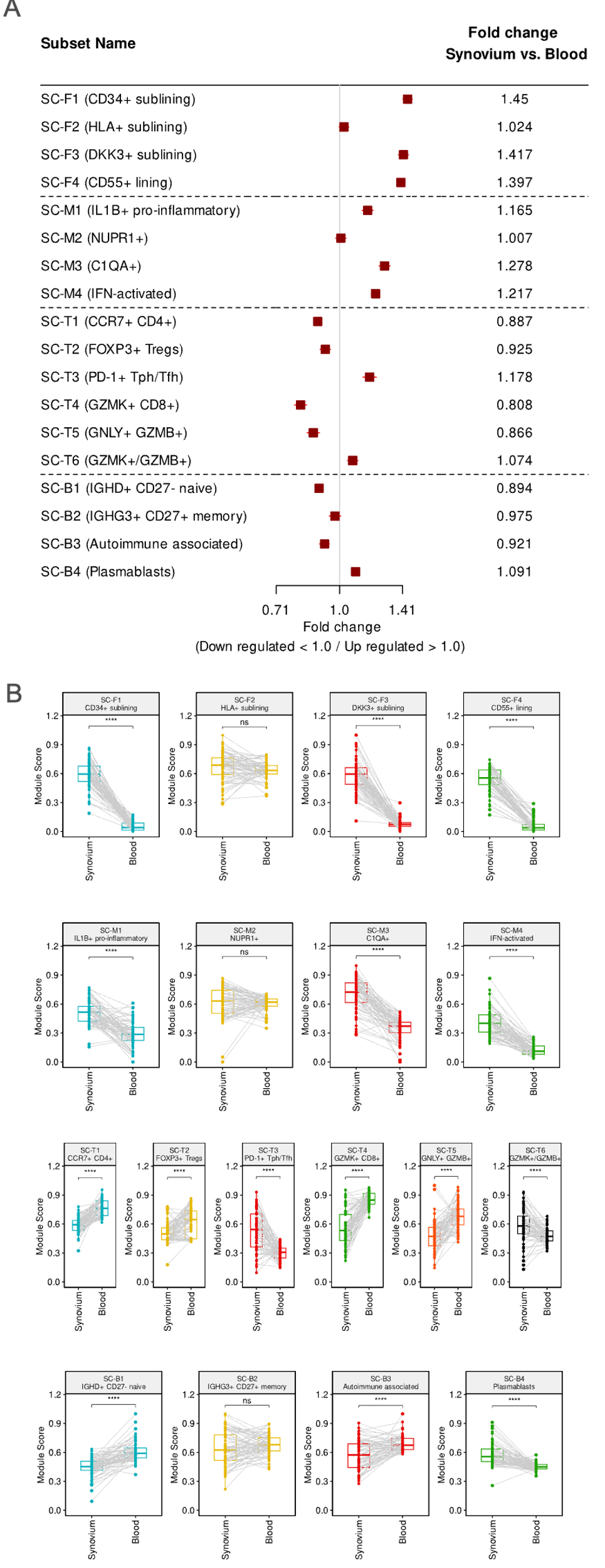
**

A, single cell state derived signatures in peripheral blood and synovia, fold changes showing on the left signatures upregulated in peripheral blood and on the right in synovia.

B, dotplots of single cell signatures in peripheral blood and synovia. Individual dots represents individual patients, boxplots show median and first and third quartiles, whiskers extending to the highest and lowest values.. * p<0.05, **p<0.01, ***p<0.001 Mann-Whitney test. n=36
